# Inspection of the Microbiota in Endodontic Lesions

**DOI:** 10.3390/dj7020047

**Published:** 2019-05-01

**Authors:** Mario Dioguardi, Giovanni Di Gioia, Gaetano Illuzzi, Claudia Arena, Vito Carlo Alberto Caponio, Giorgia Apollonia Caloro, Khrystyna Zhurakivska, Iolanda Adipietro, Giuseppe Troiano, Lorenzo Lo Muzio

**Affiliations:** 1 Department of Clinical and Experimental Medicine, University of Foggia, Via Rovelli 50, 71122 Foggia, Italy; digioia-giovanni@outlook.it (G.D.G.); gaetano.illuzzi@unifg.it (G.I.); claudia.arena@unifg.it (C.A.); vito_caponio.541096@unifg.it (V.C.A.C.); khrystyna.zhurakivska@unifg.it (K.Z.); iole.adipietro@gmail.com (I.A.); giuseppe.troiano@unifg.it (G.T.); lorenzo.lomuzio@unifg.it (L.L.M.); 2 Department of Emergency and Organ Transplantation, Nephrology, Dialysis and Transplantation Unit, University of Bari Via Piazza Giulio Cesare, 70124 Bari, Italy; giorgiacaloro1983@hotmail.it

**Keywords:** microbial endodontic, endodontic failure, endodontic bacteria, endodontic treatment

## Abstract

The primary objective of endodontic therapy is to create a biologically acceptable environment within the root canal system that allows for the healing and maintenance of the health of the peri-radicular tissue. Bacteria are one of the main causes of pulp problems, and they have different methods of penetrating and invading the endodontic space such as through carious lesions, traumatic pulp exposures, and fractures. The types of bacteria found range from facultative anaerobes to aerobes, up to the most resistant species able to survive in nutrient-free environments; the bacterial species *Enterococcus faecalis* belongs to this last group. *Enterococcus faecalis* is considered one of the main causes of recurring apical periodontal lesions following endodontic treatment, with persistent lesions occurring even after re-treatment. The review presented in this paper was performed in accordance with the PRISMA protocol and covers articles from the related scientific literature that were sourced from PubMed, Scopus, and Google Scholar using the following terms as keywords: “endodontic treatment”, “endodontic bacteria”, “microbial endodontic”, and “endodontic failure”. Only the articles considered most relevant for the purposes of this paper were read in full and taken into consideration for the following review. The results show that *Enterococcus faecalis*, *Actinomycetes*, and *Propionibacterium propionicum* are the species most frequently involved in persistent radicular and extra-radicular infections.

## 1. Introduction

In daily clinical practice, bacteria are the most common cause of lesions, which lead to pulp necrosis [1]. Although the endodontic approach remains the treatment of choice, such endodontic procedures cannot always achieve the objectives of shaping [2,3] and cleaning [4]. In addition, the lack of an apical seal [5] leads to the formation of a micro-environment [6] that is favorable to the development and selection of facultative anaerobic bacteria [7] including *Enterococcus faecalis* [8]. Numerous authors have shown that this bacterial species resists the action of sodium hypochlorite at concentrations over 5% [9].

The absence of an apical seal [10], together with the absence of a coronal seal, makes the endodontic system a perfect environment for bacteria to proliferate and form a biofilm [11]. The immune system is unable to counteract the pathogens present in the canals, and the absence of an apical seal allows the bacteria to obtain numerous nutrients via the blood vessels, while the loss of the coronal seal enables new bacteria to enter the tooth [12].

The persistence of pathologies such as chronic apical periodontitis, despite endodontic treatments, can lead to persistent intra-radicular and extra-radicular infections that are sustained by Gram-negative anaerobic bacteria such as *Actinomycetes*, *Propionibacterium propionicum*, and *Enterococcus faecalis*. Furthermore, *Enterococcus faecalis* has the ability to penetrate and invade the dentinal tubules [13]. Intracanal medicaments can be used in order to reduce the intracanal bacterial load, but they cannot effectively resolve persistent intracanal infections in the absence of an apical and coronal seal.

With acute or chronic apical periodontitis [14], antibiotics can effectively reduce inflammation [15] but are unable to eliminate it completely, since they cannot reach the bacteria present inside the canal system [16]. Therefore, the aim of this study is to identify the microbiological problems related to persistent root apical lesions that can cause therapeutic failure and lead to a loss of dental elements.

## 2. Materials and Methods

The following review was performed in accordance with the PRISMA protocol [17].

All clinical studies concerning endodontic problems and their clinical manifestations were taken into consideration. We defined the following inclusion and exclusion criteria to determine the articles’ eligibility for the qualitative analysis:

- Inclusion criteria: All scientific studies or systematic reviews investigating the bacterial associations with persistent endodontic lesions where the bacterial identification method was clearly described (PCR 16s rDNA, PCR 16s rRNA, test culture) were considered, with priority given to the most recent and updated studies. The most appropriate scientific studies on the oral microbiota in healthy tissues were identified using the most exhaustive and updated systematic revision present in the literature.
- Exclusion criteria: All articles that only considered the microbiological aspects of endodontics in a marginal way were excluded, along with case reports.

The articles were identified using electronic databases, namely PubMed and Scopus, and their bibliographies were scrupulously examined and experts in the field were consulted to identify further articles. There were no restrictions regarding the language of publication. Only articles published in the last 30 years were taken into consideration. The last search was conducted on 11 January 2019. The Ebsco and Web of Science databases were consulted, but produced results comparable to Scopus and PubMed for the keywords used. Therefore, it was decided not to increase the overlaps, and not use them.

The following keywords were entered into the PubMed and Scopus databases, with the numbers of results identified for each one indicated in brackets: “endodontic treatment” (PubMed: 4183 records, Scopus: 5225 records), “endodontic bacteria” (PubMed: 21, Scopus: 30), “microbial endodontic” (PubMed: 756, Scopus: 0), “endodontic failure” (PubMed: 157, Scopus: 290), “persistent intra-radicular infection” (PubMed: 6, Scopus: 10), and “persistent extra-radicular infection” (PubMed: 13, Scopus: 2).

The research and subsequent screening of the results obtained were conducted by two independent reviewers. A third reviewer had the task of deciding in debatable situations with a k-agreement of 0.64 (Table 1).

**Figure 1:**
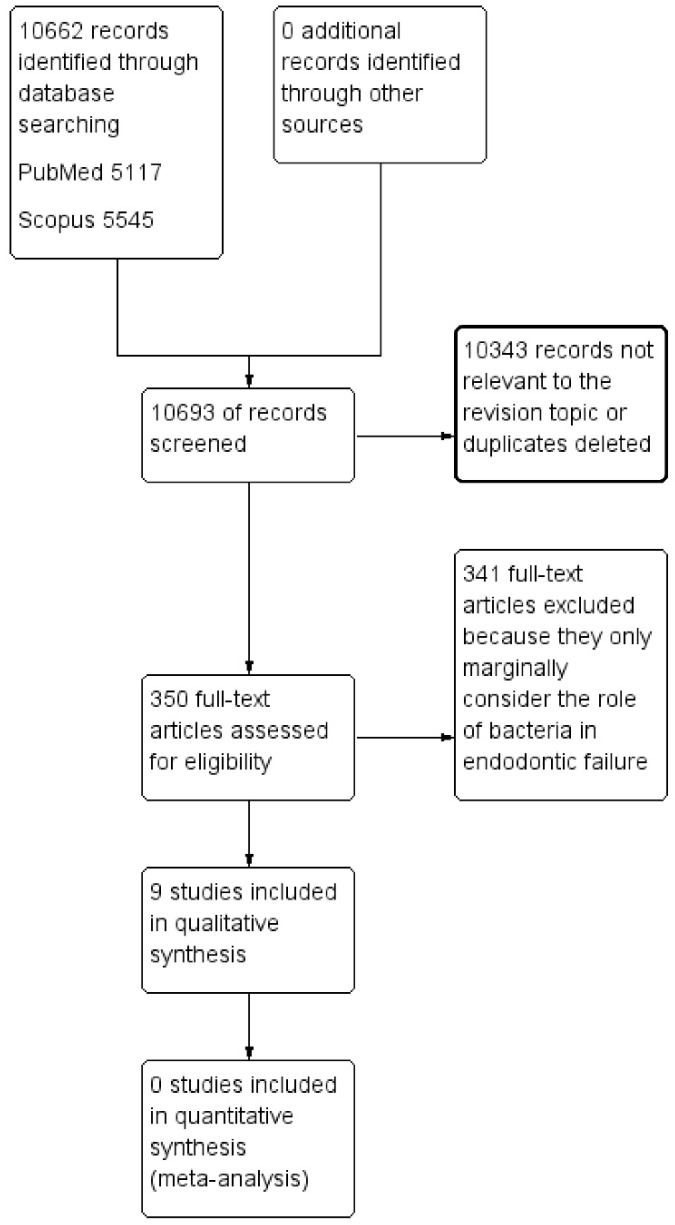
The flow chart describing the research methodology following the PRISMA protocol guidelines.

The screening phase involved a detailed analysis of the title and abstract of the resulting articles to eliminate those not relevant to the theme of the review; duplicates were then eliminated, as described in the flowchart (Figure 1).

Eligible articles were read in full to validate their appropriateness for the qualitative analysis; rejections were resolved by a fourth reviewer. The reviewers appointed to the research and screening were dentists practicing at the Department of Clinical and Experimental Medicine at the University of Foggia (Italy); the fourth reviewer, who had the task of supervision, was the director of the Department of Clinical and Experimental Medicine.

## 3. Results

A total of 10,693 records were identified from the databases (PubMed, Scopus) and are described in Table 2. The applied research methodology, the PRISMA protocol [17], is shown in the flow chart (Figure 1). The details and content of the studies taken into consideration for this paper are detailed in Table 3.

The screening performed by the two reviewers revealed an innumerable quantity of potentially eligible articles (350 records). The authors decided in agreement to include the most representative and updated studies that reflected the topic of the revision in compliance with the eligibility criteria (Table 2).

From the study of the literature, it emerges that the endodontic microbiota, in a health situation, should be absent inside the canal and the pulp, whether the dental element has been correctly treated endodontically, or if the tooth does not present pathologies. The results of the review show a series of microbial associations within the endodontic, combining the presence of specific diseases affecting pulp and apical tooth tissues.

These diseases are caries, serous and purulent pulpitis, necrosis of the pulp, and acute and chronic apical periodontitis. The microbiota varies in the presence of the different clinical conditions of the tooth and is influenced not only by the environment, but also by therapeutic treatments to solve the disease, in fact several microbiota have been identified as a function of pathologies and of the previous endodontic treatment (Table 4).

The analysis of the literature showed that a large number of scientific works in this area have focused on the microbiological aspects of persistent intra-radicular infections (Table 2). Regarding persistent extra-radicular infections, the literature is less prolific, with a decidedly lower number of scientific articles and clinical studies. The majority of these studies are case reports that did not investigate the microbiological causes of infection.

## 4. Discussion

More than 700 bacterial species were identified in the oral cavity [18], all with the potential to lead to contamination of the dental pulp and root canals [19]. In infected canals, however, there was a decidedly lower number of bacterial species, sometimes only one, which promoted the disease. The selection of bacterial species within the endodontium depends on the presence of a strong selective pressure driven by the anaerobic environment, the availability of nutrients [20], and the competition and interaction between microbial species [21].

### 4.1. Penetration and Invasion of the Endodontic Space

Penetration of bacteria inside the canals can occur through the carious process or via the dentinal tubules [22,23], for example, following dental trauma without pulp exposure [24]. The first bacteria to appear are generally facultative anaerobes or aerobes [25].

The endodontic environment has a reduced or no oxygen concentration, which promotes the development of an anaerobic bacterial structure [26]. Oxygen consumption with the production of aerobic metabolism derivatives produces a selective environment for anaerobic bacteria [27].

Another important factor in the selection of bacterial species is the availability of nutrients [28], which can be derived from connective tissues in the degenerative phase, the oral cavity, the contents of the dentinal tubules, or the fluids coming from the periapical tissues.

Endogenous substances from the periapical tissues such as glycoproteins and proteins represent the nutrient substrate for anaerobic bacteria inside the canal, while exogenous nutrients from the oral cavity affect the coronal microbial flora [29].

The first invasion phase is characterized by the presence of facultative anaerobes and aerobes, primarily represented by the bacteria belonging to the *Streptococcus* genus [30], whose main energy source is carbohydrates. In this phase, a selective pressure is established that makes the bacterial flora change course, favoring the anaerobes that can ferment amino acids and peptides [31].

In a similar way to the invasion of the enamel surfaces of the tooth, invasion of the endodontic space occurs with the formation of a biofilm that coincides with the establishment of chronic infection [32]. The biofilm provides bacteria with greater resistance to antimicrobial action [33]—compared to the planktonic form—by antibiotics, disinfectants, and detergents. Furthermore, while activating the immune response, the antibodies are unable to act effectively, since they cannot penetrate the biofilm. Antibiotic therapies solve the symptoms and signs caused by bacterial cells in the planktonic phase released by the biofilm, but they cannot completely eliminate those present in the biofilm’s deeper layers [34]. Where outbreaks of infection remain within a previously endodontically-treated root canal, immune defenses become negligible, since the pulpal microcirculation has been destroyed by inflammation or has been mechanically removed by the dentist. Therefore, any direct communication with the bloodstream is interrupted. For the same reasons, antibiotics cannot reach the area of the infectious outbreak [35].

### 4.2. Microbiological Differences between Bacterial Flora in Endodontically Treated Canals and in Untreated Canals

The presence of bacterial flora in untreated canals is regulated by the availability of nutrients and oxygen [36]. The bacteria present will be from a single species or several species of an anaerobic type, which are able to ferment peptides and amino acids. After the pulp has degraded, a considerable source of proteins is available, since the bacteria induce periapical inflammation, which causes the influx of exudate containing the aforementioned nutrients into the canal. If a dental element is treated endodontically, but with one or more canals untreated, the microbial flora present will be in a similar situation to the previous ones [37]. The bacterial associations described in the literature are the following, and concern untreated endodontic canals [38]: (*F. nucleatum*, *P. endodontalis*, *P. micos*, *C. rectus*) [39,40], (*P. intermedia*, *P. micros*, *P. anaeurobius*, *eubacteria*) [41], and (*eubacteria*, *Prevotella*, *Peptostreptococcus*) [42].

In endodontically treated root canals, all the necrotic pulp will have been eliminated, thus compromising the survival of any remaining microbial species, which are only occasionally able to find nutrients in the exudate coming from the apical or lateral periodontal tissues [43]. It is possible for a new infection of the canal to form as a result of species present in the tissues that have survived the endodontic treatment or due to the loss of the coronal seal.

Persistent diseases such as chronic apical periodontitis may be sustained by persistent infections of endodontically treated teeth [44]. To promote disease, the bacteria must not only survive but also possess the necessary properties to facilitate inflammation outside the canals [45]. In order to sustain an infection associated with chronic inflammation, the bacteria must be able to resist or protect themselves from common canal irrigants, taking refuge in the lateral canals or dentinal tubules—spaces with a greater level of protection from antimicrobial agents. In addition, dentine acts as a natural buffer to increase the pH induced by the use of calcium hydroxide-based medications [46].

Consequently, if the root canal treatment does not achieve its objectives of cleansing and shaping and the three-dimensional obturation of the canals, and if the bacteria are in communication with the exudate present in the periodontal tissues, they cannot only survive, but can also initiate the development of apical or lateral periodontal disease if they are in communication with the outside [47]. Moreover, a consequence of endodontic failure may be invasion by bacteria, from the root apex to the maxillary sinuses, giving rise to sinusitis of endodontic origin [48], the bacteria most involved in sinusitis infections are mainly of the species *Actinomyces israelii*, *Propionibacterium propionicum*, and *Enterococcus faecalis* [49]. These bacteria are also commonly associated with persistent extra-radicular infections. The ability to survive on the outer surface of the root and to involve by the contiguity maxillary sinus, may depend on the capability to aggregate in the biofilm, preventing phagocytosis by the cells of the immune system [50].

The bacteria that are most frequently responsible for the persistent diseases associated with endodontically treated teeth are mainly Gram-positive with an equal distribution of facultative and obligate anaerobes, with a prevalence of streptococci and enterococci [51], and among these, the main suspect is *Enterococcus faecalis* [52].

### 4.3. Gut Microbiome Inside the Oral Cavity. A Possible Explanation

A problem to be faced is how a bacterium such as *Enterococcus faecalis*, a species commonly found in the intestinal microbiota, can reach and colonize the internal surface of the dental elements.

We know from the literature that *Enterococcus faecalis* can survive the strenuous environmental conditions of the endodontically treated tooth. In contrast to the commensal bacteria in the oral cavity, it finds an ideal developmental environment in the necrotic tooth [25]. In fact, during apical periodontitis, enterococcus was found on apparently correctly treated dental elements and on those where the canal obturation was incomplete [53]. The use of intracanal medicaments is irrelevant for *Enterococcus faecalis*, unlike other microbial species [51].

There are two hypotheses for the presence of *Enterococcus faecalis* in the teeth: the first involves *enterococci* as primary colonizers of the canals before necrosis; the second one foresees their presence as opportunistic invaders during or following the canal treatment [54].

The first hypothesis foresees *enterococci* as probable etiologic agents of carious lesions, studies conducted by Drucker and Green (1981), Chestnutt et al. (1994), and from Gold et al. (1975) demonstrated the ability of *Enterococcus faecalis* to generate carious lesions even if the persistence of the enterococcus strains in the oral cavity was only a few weeks [55,56,57]. *Enterococci* have a low cariogenic capacity and more recent studies such as that by Chun et al. (2005) demonstrated that the presence of *Enterococcus faecalis* in sites with active caries was 10 times lower than other bacteria commonly associated with caries (*Streoptococcus mutans*), making the unlikely penetration of the latter into the pulp as the primary colonizer [58]. Other ways of *Enterococcus faecalis* penetration inside the tooth can be fissures and cracked enamel, moreover, it also contemplated the possibility of the contamination of necrotic teeth by anacoresis through the bloodstream, starting from the gut intestine [59].

The second hypothesis foresees a subsequent contamination of the tooth during root canal treatment or following infiltration of the coronal seal.

For *Enterococcus faecalis* during root canal treatment, in fact, a study conducted by [60] demonstrates how *Enterococcus faecalis*, once it has infected the canal, has a greater ability to resist endodontic treatments in comparison with accidental contamination during treatment. Another way of penetrating the *enterococci* during the treatment could be through the infiltration of the temporary obturation, even if the evidence comes from a few studies, it is more probable that the infection comes from the presence of permanent restorations infiltrated after the treatment [61].

*Enterococcus faecalis* is not a commensal of the oral cavity and is only occasionally found; in fact it is notoriously present in the gastrointestinal tract, colonizing niches of oral mucosa only for short periods where it is possible to find it. The presence of *enterococci* in the oral cavity probably comes from contamination following ingestion of contaminated food. *Enterococci* are practically ubiquitous on the surface of food, especially in dairy milks and cured meats, and are also present in foods such as vegetables, fish, seafood, and meat. Contamination of the oral cavity therefore probably occurs through food and is compatible with innumerable studies that have found the presence of *Enterococcus faecalis* only occasionally in the mouth, and especially after the ingestion of food contaminated by *Enterococcus faecalis* [62].

Therefore, we can establish that the main way of *Enterococcus faecalis* penetration is in the canals, after the intake of contaminated food, and after endodontic treatment by infiltration of the coronal restoration after endodontic treatment.

### 4.4. The Role of Enterococcus Faecalis in Chronic Apical Periodontitis

*Enterococci* are Gram-positive bacteria that are part of the commensal flora of the gastrointestinal tract and are arranged in short chains or pairs and widely distributed in the environment.

*Enterococci* involved in infections of certain clinical importance are *Enterococcus faecalis* and *Enterococcus faecium*. The virulent species, *Enterococcus faecalis*, which is responsible for 80% of human infections [63], is capable of resistance in extreme conditions, can multiply at temperatures between 10 and 45 °C, and can withstand an alkaline pH of 10 hyperosmolar NaOCl solutions at a concentration of 6.5% [64]; it can also withstand hydrogen peroxide, ethanol, sodium hypochlorite, and ultraviolet radiation in the absence of nutrients for prolonged periods of time [9].

In the endodontic environment, the *enterococci* species has the ability to colonize the dentinal tubules deeply, creating an extensive biofilm and metabolizing the tubules’ collagen fibers in the event of nutrient reduction [65]. Regarding the virulence factors, it is also important to mention their ability to produce aggregating substances, adhesion proteins (type 1 collagen, dentine component), and lithic enzymes such as hyaluronidase, cytosolin, serine protease, and gelatinase [66].

*Enterococcus faecalis* has the capacity to sustain an inflammatory state by modulating its intensity and inhibiting the leukocyte response; it can also draw nourishment from the serum proteins, which come from the canal through the improperly sealed apical foramen [54].

This species is not part of the oral commensal flora, but is occasionally found in the oral cavity following the ingestion of contaminated food. The mechanism by which the *Enterococcus faecalis* species succeeds in penetrating the endodontic system is not yet fully understood. Possible methods of penetration include through the front of a carious lesion in contiguity with the endodontic system in a state of necrosis or inflammation; through dentinal fractures, or due to a lack or loss of the coronal seal after endodontic treatment; through lateral canals or root fractures; or by entering through the bloodstream (hypothesis contemplated, but yet to be demonstrated) [67].

The scientific literature is in agreement that *Enterococcus faecalis* is not among the main causes of primary endodontic infections, but causes one of the most recurrent and persistent forms of chronic apical periodontitis [68].

Its ability to resist disinfectants, invade dentinal tubules [32], withstand long periods of food deprivation, and create an inflammatory response certainly make this bacterium a tough enemy to defeat.

Re-treatment in situations of endodontic failure is always a valid alternative to extraction or the disproportionate administration of antibiotics, which are only useful for acute inflammatory states [69].

In cases where chronic apical periodontitis persists even after re-treatment, even if performed correctly, one might not consider persistent intra-radicular infection [70], but rather, persistent apical extra-radicular infection [71,72]. Such infections are no longer etiologically sustained by bacteria, but by *Actinomycetes* such as *Actinomyces israelii* (the most frequent) [73], and in some cases, *Propionibacterium propionicum* [74].

**Figure 2:**
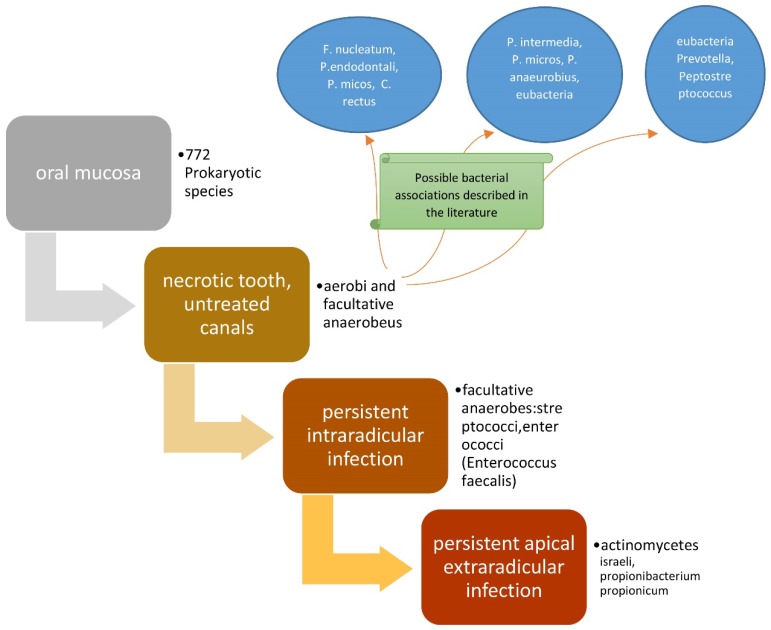
Scheme representing the roles of bacteria in endodontic diseases.

In these clinical situations, it is possible to preserve the dental element by performing an apectomy of the roots concerned, whenever possible; otherwise, it is necessary to extract the dental element [71] (Figure 2).

## 5. Conclusions

Pulp diseases are the main cause of the invasion of endodontic spaces by oral microbial flora. The survival of bacteria and fungi in this environment depends on a series of factors that involve the presence of nutrients, the environmental conditions of anaerobiosis, the pH value, competition/cooperation with other microorganisms, and the microenvironmental characteristics. The environment determines which microorganisms survive, and some can survive under unfavorable conditions. In order to discern between cases of intra-radicular and persistent extra-radicular infection, the endodontist must pay attention to the clinical signs and symptoms in the diagnostic phase that allow for the pathology to be correctly identified. This allows the endodontist to intercept, in the preliminary phase, all situations involving apical periodontal inflammation, both acute and chronic [75]. The effectiveness of endodontic re-treatment involving suitable antibiotic therapy [76] is not conclusive, even if correct shaping and a complete root canal obturation according to dental standards has been undertaken. Other possible treatments that the dentist can adopt are an apicectomy, or, as a last resort, extraction of the dental element.

We can summarize the conclusions of the review in the following points:

1. Several microbes can be identified depending on the health status of the tooth (caries, pulpitis, necrotic tooth, acute/chronic parodontitis, persistent extra/intra-radicular infections);
2. In endodontic failures, the most involved bacterial species are facultative anaerobes with predominance in recurrent forms of apical periodontitis of *Enterococcus faecalis*;
3. In persistent extra-radicular infections, the bacteria mainly involved are *A. israeli* and *Propionibacterium propionicum*;
4. The presence of intestinal microbiome in the oral cavity and consequently inside the tooth is due to food contamination by *enterococci*.

## Figures and Tables

**Table 1:** K-agreement calculation, Po = 0.8914, Pe = 0.695, K-agreement = 0.644 (<0 no agreement, 0.0–0.20 slight agreement, 0.21–0.40 fair agreement, 0.41–0.60 moderate agreement, 0.61–0.80 substantial agreement, 0.81–1.00 almost perfect agreement).

		Reviewer 2	Reviewer 2	Reviewer 2	
		Include	Exclude	Unsure	Total
**Reviewer 1**	**Include**	9	0	0	9
**Reviewer 1**	**Exclude**	3	268	10	281
**Reviewer 1**	**Unsure**	0	25	35	60
	**Total**	12	293	45	350

**Table 2:** A complete overview of the search methodology, illustrating the keywords used, the Boolean operators adopted, and the number of records obtained for each online database.

Keywords	PubMed	Scopus
Persistent intra-radicular infection	6	10
Persistent extra-radicular infection	13	2
Endodontic treatment	4183	5225
Endodontic bacteria	21	30
Microbial endodontic	756	0
Endodontic failure	157	290
Total number of records	5136	5557

**Table 3:** The complete list of the nine articles eligible for the qualitative analysis with descriptions of their topics and results. The nine articles were selected from 350 potentially eligible articles and selected by two different reviewers. The choice of these nine articles reflects the inclusion and exclusion criteria. The most up-to-date and relevant articles for the topic of interest of the review were chosen, articles in doubt for the individual reviewer or in disagreement were excluded from the table.

First Author, Date	Journal	Type of Study	Endodontic Diseases Associated with Bacteria Examined in the Study	Main Bacteria That Are Associated with Pathology Examined in the Study	Characteristics of the Main Bacteria Taken into Consideration	Identification Method
Verma, D., 2018	Archives of microbiology	Review (data: The expanded Human Oral Microbiome Database)	Normal oral cavity	772 prokaryotic species (96% six broad phyla: Firmicutes, Actinobacteria, Proteobacteria, Fusobacteria, Bacteroidetes, and Spirochaetes)	Gram-positive, Gram-negative, anaerobic and aerobic.	PCR 16S rDNA
Shin, J.M., 2018	Journal of endodontics	Review (data: PubMed—Next Generation Sequencing Applications)	Necrotic teeth in untreated canals, pulpitis, primary endodontic infections	*Eubacteria (Firmicutes, Actinobacteria, Proteobacteria, Fusobacteria, Bacteroidetes, and Spirochaete)* *Prevotella intermedia* *Peptostreptococcus* *Fusobacterium* *Porphyromonas* *Parvimonas* *Streptococcus*	Gram-positive, Gram-negative, anaerobic and aerobic Gram-negative, obligate anaerobic Gram-positive, anaerobic Gram-positive anaerobic Gram-negative, anaerobic Gram-positive, aerobic Gram-positive, anaerobes;	PCR 16S rRNA
Guven, Y., 2018	Indian journal of dental research: Official publication of the Indian Society for Dental Research	Clinical study	Necrotic teeth, abscessed primary teeth, primary endodontic infections	*Prevotella intermedia* *Peptostreptococcus micros* *Peptostreptococcus anaeurobius* *Eubacteria* *Fusobacterium nucleatum*	Gram-negative, obligate anaerobic; Gram-positive anaerobic; Gram-positive, anaerobic; Gram-positive, Gram-negative, anaerobic and aerobic Gram-negative, anaerobic	PCR 16S rRNA
Siqueira, J.F., 2003	Journal of endodontics	Clinical study	The necrotic tooth in untreated canals, primary endodontic infections	*Fusobacterium nucleatum* *Porphyromonas endodontalis* *Peptostreptococcus micros* *Campylobacter rectus* *Dialister pneumosintes*	Gram-negative, anaerobic Gram-negative, anaerobic Gram-positive anaerobic Gram negative, facultatively anaerobic Gram-negative, anaerobic	PCR 16S rDNA
Siqueira, J.F., 2002	Journal of endodontics	Clinical study	Necrotic teeth in untreated canals, primary endodontic infections	*Actinomyces species* *Streptococci* *Enterococcus faecalis*	Gram-positive, anaerobes; Gram-positive, facultative anaerobes Gram-positive, facultative anaerobes	PCR 16S rRNA
Love, R.M., 2001	International endodontic journal	Clinical study	Persistent intra-radicular infection	*Enterococcus faecalis*	Gram-positive, facultative anaerobic	Culture
Molande, A., 1998	International endodontic journal	Clinical study	Persistent intra-radicular infection	*Streptococci* *Enterococci (Enterococcus faecalis)*	Gram-positive, facultative anaerobes Gram-positive, facultative anaerobes	Culture
Xia, T., 2003	Journal of endodontics	Clinical study	Persistent apical extra-radicular infection	*Actinomycetes (A. israeli)*	Gram-positive, anaerobic.	PCR 16S rRNA
Grgurevic, J., 2017	Acta Stomatol Croat	Clinical study	Persistent apical extra-radicular infection	*Propionibacterium propionicum*	Gram-positive, anaerobic.	PCR 16S rRNA

**Table 4:** Microbiota found in the literature.

Environment or Pathology	Type of Bacteria	Prevalent Bacteria That to Form the Microbiota
**Oral mucosa**	Gram-positive, Gram-negative, anaerobic and aerobic	772 Prokaryotic species
**Necrotic tooth, Untreated canals**	Gram-positive, Gram-negative, anaerobic and aerobic	*Eubacteria* *Prevotella intermedia* *Peptostreptococcus* *Fusobacterium* *Porphyromonas* *Parvimonas* *Streptococcus* *Campylobacter rectus* *Dialister pneumosintes* *Actinomyces species* *Enterococcus faecalis*
**Persistent intra-radicular infection**	Gram-positive, facultative anaerobes	*Streptococci* *Enterococci (Enterococcus faecalis*)
**Persistent extra-radicular infection**	Gram-positive, anaerobic	*Actinomycetes (A. israeli)* *Propionibacterium propionicum*

## References

[1] Saito D., Leonardo Rde T., Rodrigues J.L., Tsai S.M., Hofling J.F., Goncalves R.B. (2006). Identification of bacteria in endodontic infections by sequence analysis of 16S rDNA clone libraries. J. Med. Microbiol..

[2] Dioguardi M., Troiano G., Laino L., Lo Russo L., Giannatempo G., Lauritano F., Cicciu M., Lo Muzio L. (2015). ProTaper and WaveOne systems three-dimensional comparison of device parameters after the shaping technique. A micro-CT study on simulated root canals. Int. J. Clin. Exp. Med..

[3] Troiano G., Dioguardi M., Cocco A., Zhurakivska K., Ciavarella D., Muzio L.L. (2018). Increase the glyde path diameter improves the centering ability of F6 Skytaper. Eur. J. Dent..

[4] Troiano G., Dioguardi M., Cocco A., Giannatempo G., Laino L., Ciavarella D., Berutti E., Lo Muzio L. (2016). Influence of Operator’s Experience on the Shaping Ability of Protaper Universal and Waveone Systems: A Comparative Study on Simulated Root Canals. Open Dent. J..

[5] Buonavoglia A., Lauritano D., Perrone D., Ardito F., Troiano G., Dioguardi M., Candotto V., Silvestre F.J., Lo Muzio L. (2017). Evaluation of chemical-physical properties and cytocompatibility of TheraCal LC. J. Biol. Regul. Homeost. Agents.

[6] Troiano G., Perrone D., Dioguardi M., Buonavoglia A., Ardito F., Lo Muzio L. (2018). In vitro evaluation of the cytotoxic activity of three epoxy resin-based endodontic sealers. Dent. Mater. J..

[7] Assed S., Ito I.Y., Leonardo M.R., Silva L.A., Lopatin D.E. (1996). Anaerobic microorganisms in root canals of human teeth with chronic apical periodontitis detected by indirect immunofluorescence. Endod. Dent. Traumatol..

[8] Swimberghe R.C.D., Coenye T., De Moor R.J.G., Meire M.A. (2018). Biofilm model systems for root canal disinfection: A literature review. Int. Endod. J..

[9] Dioguardi M., Di Gioia G., Illuzzi G., Laneve E., Cocco A., Troiano G. (2018). Endodontic irrigants: Different methods to improve efficacy and related problems. Eur. J. Dent..

[10] Dioguardi M., Perrone D., Troiano G., Laino L., Ardito F., Lauritano F., Cicciu M., Muzio L.L. (2015). Cytotoxicity evaluation of five different dual-cured resin cements used for fiber posts cementation. Int. J. Clin. Exp. Med..

[11] Chevalier M., Ranque S., Precheur I. (2018). Oral fungal-bacterial biofilm models in vitro: A review. Med. Mycol..

[12] Ducret M., Fabre H., Celle A., Mallein-Gerin F., Perrier-Groult E., Alliot-Licht B., Farges J.C. (2017). Current challenges in human tooth revitalization. Biomed. Mater. Eng..

[13] Haapasalo M., Orstavik D. (1987). In vitro infection and disinfection of dentinal tubules. J. Dent. Res..

[14] Braz-Silva P.H., Bergamini M.L., Mardegan A.P., De Rosa C.S., Hasseus B., Jonasson P. (2019). Inflammatory profile of chronic apical periodontitis: A literature review. Acta Odontol. Scand..

[15] Parhizkar A., Nojehdehian H., Asgary S. (2018). Triple antibiotic paste: Momentous roles and applications in endodontics: A review. Restor. Dent. Endod..

[16] Trusewicz M., Buczkowska-Radlinska J., Giedrys-Kalemba S. (2005). The effectiveness of some methods in eliminating bacteria from the root canal of a tooth with chronic apical periodontitis. Ann. Acad. Med. Stetin..

[17] Liberati A., Altman D.G., Tetzlaff J., Mulrow C., Gotzsche P.C., Ioannidis J.P., Clarke M., Devereaux P.J., Kleijnen J., Moher D. (2009). The PRISMA statement for reporting systematic reviews and meta-analyses of studies that evaluate health care interventions: Explanation and elaboration. PLoS Med..

[18] Verma D., Garg P.K., Dubey A.K. (2018). Insights into the human oral microbiome. Arch. Microbiol..

[19] Hirai K., Tagami A., Okuda K. (1991). Isolation and classification of anaerobic bacteria from pulp cavities of nonvital teeth in man. Bull. Tokyo Dent. Coll..

[20] Grenier D., Mayrand D. (1986). Nutritional relationships between oral bacteria. Infect. Immun..

[21] Foster K.R., Bell T. (2012). Competition, not cooperation, dominates interactions among culturable microbial species. Curr. Biol..

[22] Kwang S., Abbott P. (2014). The presence and distribution of bacteria in dentinal tubules of root filled teeth. Int. Endod. J..

[23] Love R.M., Jenkinson H.F. (2002). Invasion of dentinal tubules by oral bacteria. Crit. Rev. Oral Biol. Med..

[24] Ricucci D., Siqueira J.F., Loghin S., Berman L.H. (2015). The cracked tooth: Histopathologic and histobacteriologic aspects. J. Endod..

[25] Fabricius L., Dahlen G., Ohman A.E., Moller A.J. (1982). Predominant indigenous oral bacteria isolated from infected root canals after varied times of closure. Scand. J. Dent. Res..

[26] Sundqvist G. (1994). Taxonomy, ecology, and pathogenicity of the root canal flora. Oral Surg. Oral Med. Oral Pathol..

[27] Siqueira J.F., Rocas I.N., Alves F.R., Silva M.G. (2009). Bacteria in the apical root canal of teeth with primary apical periodontitis. Oral Surg. Oral Med. Oral Pathol. Oral Radiol. Endod..

[28] Chavez de Paz L.E., Hamilton I.R., Svensater G. (2008). Oral bacteria in biofilms exhibit slow reactivation from nutrient deprivation. Microbiology.

[29] Ingar O. (2009). Open access to oral microbiology. J. Oral Microbiol..

[30] Fouad A.F., Kum K.Y., Clawson M.L., Barry J., Abenoja C., Zhu Q., Caimano M., Radolf J.D. (2003). Molecular characterization of the presence of Eubacterium spp and Streptococcus spp in endodontic infections. Oral Microbiol. Immunol..

[31] Narayanan L.L., Vaishnavi C. (2010). Endodontic microbiology. J. Conserv. Dent..

[32] Love R.M. (2002). Bacterial adhesins—Their role in tubule invasion and endodontic disease. Aust. Endod. J..

[33] Venkatesan N., Perumal G., Doble M. (2015). Bacterial resistance in biofilm-associated bacteria. Future Microbiol..

[34] Al-Ahmad A., Ameen H., Pelz K., Karygianni L., Wittmer A., Anderson A.C., Spitzmuller B., Hellwig E. (2014). Antibiotic resistance and capacity for biofilm formation of different bacteria isolated from endodontic infections associated with root-filled teeth. J. Endod..

[35] Raslan N., Mansour O., Assfoura L. (2017). Evaluation of antibiotic mix in Non-instrumentation Endodontic Treatment of necrotic primary molars. Eur. J. Paediatr. Dent..

[36] Lee L.W., Lee Y.L., Hsiao S.H., Lin H.P. (2017). Bacteria in the apical root canals of teeth with apical periodontitis. J. Formos. Med. Assoc..

[37] Gomes B.P., Drucker D.B., Lilley J.D. (1994). Positive and negative associations between bacterial species in dental root canals. Microbios.

[38] Siqueira J.F., Rocas I.N., Souto R., de Uzeda M., Colombo A.P. (2002). Actinomyces species, streptococci, and Enterococcus faecalis in primary root canal infections. J. Endod..

[39] Ruviere D.B., Leonardo M.R., da Silva L.A., Ito I.Y., Nelson-Filho P. (2007). Assessment of the microbiota in root canals of human primary teeth by checkerboard DNA-DNA hybridization. J. Dent. Child..

[40] Siqueira J.F., Rocas I.N. (2003). Positive and negative bacterial associations involving Dialister pneumosintes in primary endodontic infections. J. Endod..

[41] Guven Y., Ustun N., Aksakal S.D., Topcuoglu N., Aktoren O., Kulekci G. (2018). Assessment of the endodontic microbiota of abscessed primary teeth using microarray technology. Indian J. Dent. Res..

[42] Shin J.M., Luo T., Lee K.H., Guerreiro D., Botero T.M., McDonald N.J., Rickard A.H. (2018). Deciphering Endodontic Microbial Communities by Next-generation Sequencing. J. Endod..

[43] Cheung G.S., Ho M.W. (2001). Microbial flora of root canal-treated teeth associated with asymptomatic periapical radiolucent lesions. Oral Microbiol. Immunol..

[44] Adib V., Spratt D., Ng Y.L., Gulabivala K. (2004). Cultivable microbial flora associated with persistent periapical disease and coronal leakage after root canal treatment: A preliminary study. Int. Endod. J..

[45] Sanchez-Sanhueza G., Bello-Toledo H., Gonzalez-Rocha G., Goncalves A.T., Valenzuela V., Gallardo-Escarate C. (2018). Metagenomic study of bacterial microbiota in persistent endodontic infections using Next-generation sequencing. Int. Endod. J..

[46] Amaral S.F.D., Scaffa P.M.C., Rodrigues R.D.S., Nesadal D., Marques M.M., Nogueira F.N., Sobral M.A.P. (2018). Dynamic Influence of pH on Metalloproteinase Activity in Human Coronal and Radicular Dentin. Caries Res..

[47] Ricucci D., Siqueira J.F. (2010). Fate of the tissue in lateral canals and apical ramifications in response to pathologic conditions and treatment procedures. J. Endod..

[48] Heling I., Rotstein I. (1989). A persistent oronasal sinus tract of endodontic origin. J. Endod..

[49] Taschieri S., Torretta S., Corbella S., Del Fabbro M., Francetti L., Lolato A., Capaccio P. (2017). Pathophysiology of sinusitis of odontogenic origin. J. Investig. Clin. Dent..

[50] Brook I. (2006). Sinusitis of odontogenic origin. Otolaryngol. Head Neck Surg..

[51] Molander A., Reit C., Dahlen G., Kvist T. (1998). Microbiological status of root-filled teeth with apical periodontitis. Int. Endod. J..

[52] Love R.M. (2001). Enterococcus faecalis—A mechanism for its role in endodontic failure. Int. Endod. J..

[53] Sundqvist G., Figdor D., Persson S., Sjogren U. (1998). Microbiologic analysis of teeth with failed endodontic treatment and the outcome of conservative re-treatment. Oral Surg. Oral Med. Oral Pathol. Oral Radiol. Endod..

[54] Zehnder M., Guggenheim B. (2009). The mysterious appearance of enterococci in filled root canals. Int. Endod. J..

[55] Drucker D.B., Green R.M. (1981). The relative cariogenicity of different streptococci in the gnotobiotic WAG/RIJ rat. Arch. Oral Biol..

[56] Chestnutt I.G., MacFarlane T.W., Stephen K.W. (1994). An in vitro investigation of the cariogenic potential of oral streptococci. Arch. Oral Biol..

[57] Gold O.G., Jordan H.V., van Houte J. (1975). The prevalence of enterococci in the human mouth and their pathogenicity in animal models. Arch. Oral Biol..

[58] Chu F.C., Tsang C.S., Chow T.W., Samaranayake L.P. (2005). Identification of cultivable microorganisms from primary endodontic infections with exposed and unexposed pulp space. J. Endod..

[59] Tziafas D. (1989). Experimental bacterial anachoresis in dog dental pulps capped with calcium hydroxide. J. Endod..

[60] Kaufman B., Spangberg L., Barry J., Fouad A.F. (2005). Enterococcus spp. in endodontically treated teeth with and without periradicular lesions. J. Endod..

[61] Sjogren U., Figdor D., Spangberg L., Sundqvist G. (1991). The antimicrobial effect of calcium hydroxide as a short-term intracanal dressing. Int. Endod. J..

[62] Franz C.M., Stiles M.E., Schleifer K.H., Holzapfel W.H. (2003). Enterococci in foods—A conundrum for food safety. Int. J. Food Microbiol..

[63] Fisher K., Phillips C. (2009). The ecology, epidemiology and virulence of Enterococcus. Microbiology.

[64] Estrela C., Silva J.A., de Alencar A.H., Leles C.R., Decurcio D.A. (2008). Efficacy of sodium hypochlorite and chlorhexidine against Enterococcus faecalis—A systematic review. J. Appl. Oral Sci..

[65] Marashdeh M.Q., Gitalis R., Levesque C., Finer Y. (2019). Endodontic pathogens possess collagenolytic properties that degrade human dentine collagen matrix. Int. Endod. J..

[66] Kayaoglu G., Orstavik D. (2004). Virulence factors of Enterococcus faecalis: Relationship to endodontic disease. Crit. Rev. Oral Biol. Med..

[67] Gutmann J.L., Manjarres V. (2018). Historical and Contemporary Perspectives on the Microbiological Aspects of Endodontics. Dent. J..

[68] Stuart C.H., Schwartz S.A., Beeson T.J., Owatz C.B. (2006). Enterococcus faecalis: Its role in root canal treatment failure and current concepts in retreatment. J. Endod..

[69] Chercoles-Ruiz A., Sanchez-Torres A., Gay-Escoda C. (2017). Endodontics, Endodontic Retreatment, and Apical Surgery Versus Tooth Extraction and Implant Placement: A Systematic Review. J. Endod..

[70] Zhang C., Du J., Peng Z. (2015). Correlation between Enterococcus faecalis and Persistent Intraradicular Infection Compared with Primary Intraradicular Infection: A Systematic Review. J. Endod..

[71] Signoretti F.G., Endo M.S., Gomes B.P., Montagner F., Tosello F.B., Jacinto R.C. (2011). Persistent extraradicular infection in root-filled asymptomatic human tooth: Scanning electron microscopic analysis and microbial investigation after apical microsurgery. J. Endod..

[72] Ricucci D., Candeiro G.T., Bugea C., Siqueira J.F. (2016). Complex Apical Intraradicular Infection and Extraradicular Mineralized Biofilms as the Cause of Wet Canals and Treatment Failure: Report of 2 Cases. J. Endod..

[73] Xia T., Baumgartner J.C. (2003). Occurrence of Actinomyces in infections of endodontic origin. J. Endod..

[74] Grgurevic J., Ivanisevic Malcic A., Tambic Andrasevic A., Prpic Mehicic G., Kuzmac S., Jukic S. (2017). Frequency of bacetrial content finding in persistant periapical lesions. Acta Stomatol. Croat..

[75] Ricucci D., Lopes W.S.P., Loghin S., Rocas I.N., Siqueira J.F. (2018). Large Bacterial Floc Causing an Independent Extraradicular Infection and Posttreatment Apical Periodontitis: A Case Report. J. Endod..

[76] Rochd T., Calas P., Jabri M., Roques C. (2010). Resistance to B-lactamines of bacteria responsible for endodontic root canal infections. Odontostomatol. Trop..

